# Effects of palm oil consumption on biomarkers of glucose metabolism: A systematic review

**DOI:** 10.1371/journal.pone.0220877

**Published:** 2019-08-15

**Authors:** Siti Hafizah Zulkiply, Vimala Balasubramaniam, Nur Ain Abu Bakar, Aswir Abd Rashed, Sophia Rasheeqa Ismail

**Affiliations:** Cardiovascular, Diabetes and Nutrition Centre, Institute for Medical Research, Ministry of Health Malaysia, Selangor, Malaysia; CUNY School of Public Health, UNITED STATES

## Abstract

**Introduction:**

Vegetable oil is an important source of fatty acids, and as palm oil being the highest consumed vegetable oil in many countries, its high saturated fatty acid content has led many concerns on cardiometabolic health. Dietary fatty acids has also been linked to affect glucose metabolism and insulin sensitivity. This systematic review is aimed at critically evaluating the available evidence on the association of palm oil with the biomarkers of glucose metabolism as compared to other vegetable oils.

**Methods:**

We systemically searched PubMed, CENTRAL and Scopus up to June 2018. We searched for published interventional studies on biomarkers of glucose metabolism (defined as fasting glucose, fasting insulin, HOMA, 2-hour post prandial glucose and HbA1C) that compared palm oil- or palm olein-rich diets with other edible vegetable oils (such as olive oil, canola oil and soybean oil). Two reviewers independently extracted data and assessed study risks of bias. Mean differences of outcomes were pooled for the meta-analysis.

**Results:**

We identified 1921 potentially eligible articles with only eight included studies. Seven randomised cross-over trials and one parallel trial were included. Study population were among young to middle-aged, healthy, non-diabetic, and normal weight participants. Intervention duration ranged from three to seven weeks, and fat substitution ranged from 15% to 20% energy. There were insignificant differences in fasting glucose when compared to partially hydrogenated soybean oil [-0.15mmol/L (-0.46,0.16) P = 0.33, I^2^ = 48%], soybean oil [0.05mmol/L (-0.09,0.18) P = 0.49, I^2^ = 0%] and olive oil [0.04mmol/L (-0.09,0.17) P = 0.76, I^2^ = 0%]. Insignificant effects were also seen on fasting insulin when compared to partially hydrogenated soybean oil [1.72pmol/L (-11.39,14.84) P = 0.80, I^2^ = 12%] and olive oil diet [-0.14pmol/L (-4.87,4.59) P = 0.95, I^2^ = 0%].

**Conclusion:**

Current evidence on the effects of palm oil consumption on biomarkers of glucose metabolism is poor and limited to only healthy participants. We conclude that little or no additional benefit will be obtained by replacing palm oil with other oils rich in mono or polyunsaturated fatty acids for changes in glucose metabolism.

## 1. Introduction

Historically, reduction in consumption of dietary fats have been recommended. Trials have shown that diets higher in healthful fats, exceeding 35% limit of calories from dietary fat, can reduce the risk of diabetes [1]. There is also evidence stating that the healthiest traditional diets in the world are rich in fats from vegetable oils, nuts and seafood [1]. Palm oil is the highest consumed and produced vegetable oil in the world, leading to it being a major source of saturated fat in Asia, Africa and Europe [2]. Despite its popularity in household cooking and in the food industry, palm oil has been labelled as an unhealthy fat in view of its high percentage of saturated fat as compared to other edible vegetable oils. Derived from the fruit of the palm *Elaeis guineensis*, palm oil typically contains 40% oleic acid (monounsaturated fatty acid (MUFA)), 10% linoleic acid (polyunsaturated fatty acid (PUFA)), 45% palmitic acid and 5% stearic acid (saturated fatty acid (SFA)) [3]. Owing to its high percentage of saturated fat and the historically linked association of saturated fat consumption and risk of cardiometabolic diseases [4], there have been many views and controversial opinions with regards to the adverse health effects of palm oil consumption, especially in coronary heart disease [5].

Insulin resistance is a precursor of type 2 diabetes and it is a multi-faceted disruption of the communication between insulin and the interior of a target cell [6]. A major contributor to the development of insulin resistance is an overflowing of circulating fatty acids [7,8]. Studies have shown that total fat, saturated fat, and high caloric intakes leading to obesity appeared to have detrimental effects through possibly glucose-insulin homeostasis, oxidative stress, inflammation, adipocyte metabolism and metabolic expenditure [1,9]. Dietary fats have also been shown to affect glucose metabolism by altering cell membrane function, enzyme activity, insulin signalling and gene expression [10]. Consumption of energy-dense/high fat diets is strongly and positively associated with overweight that, in turn, deteriorates insulin sensitivity, particularly when the excess of body fat is located in abdominal region [11]. While there are contrary arguments linking saturated fat intake and cardiovascular health [12–14], the association between saturated fat intake and type 2 diabetes mellitus remains unclear [15,16]. Despite these uncertainties, dietary recommendations have limited the intake of saturated fats in diabetics. Dietary guidelines on macronutrient intake for management and prevention of type 2 diabetes generally recommend increasing foods rich in MUFA and reducing SFA [17]. Additionally, diabetic patients are recommended to consume saturated fats totalling to less than 10% of their daily energy intake [18]. Palm oil is favored over other vegetable oils because of its higher melting point and resistance to oxidative changes resulting from its higher content of SFAs [19]. Therefore, industries have produced partially hydrogenated oils through hydrogenation process to extend the shell life of the fat [20]. However, this process formed trans-fat, which are no longer considered as safe (GRAS) additives by the Food and Drug Administration (FDA) due to rising evidences of harmful effects of trans-fatty acids (TFA) on health, especially towards cardiovascular diseases (CVD) [21].

To date, most of the studies examined the effect of palm oil or palmitic acid on biomarkers of CVD [12,22]. There have been only limited studies linking consumption of vegetable oils to type 2 diabetes. Hence, this review is aimed at critically evaluating the available evidence on the association of palm oil with the biomarkers of glucose metabolism as compared to other vegetable oils.

## 2. Materials and methods

**Fig 1:**
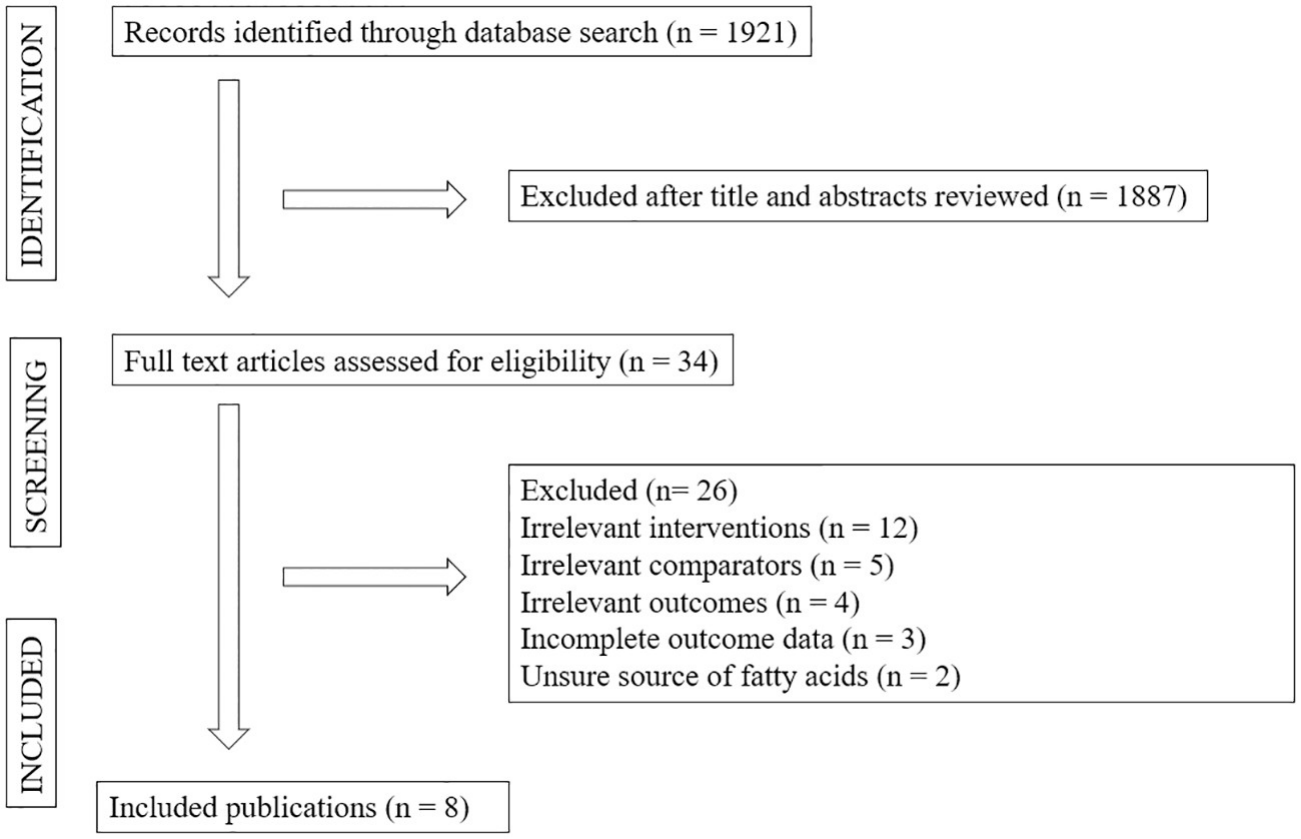
PRISMA flowchart for the selection of studies. Outcomes of the systematic review of the literature by record identification, screening, and analysis in the Preferred Reporting Items for Systematic Reviews and Meta-Analyses (PRISMA) statement flow diagram.

We conducted and reported this review in accordance to the Preferred Reporting Items for Systematic Reviews and Meta-Analyses (PRISMA) guidelines [23] and based on the Cochrane Collaboration approach [24]. The PRISMA Checklist is found in Fig 1.

### 2.1 Eligibility criteria

We included published intervention studies (defined as randomised controlled trial, crossover study and quasi-experimental study) comparing markers of glucose metabolism (fasting glucose, 2-hour post prandial glucose, hemoglobin A1C (HbA1c), homeostatic model assessment-insulin resistance (HOMA) and fasting insulin) [25,26] for palm oil consumption (defined as consumption of palm oil- or palm olein-rich diet) and other vegetable oils (such as olive oil, canola oil and sunflower oil). We included only human studies with adult participants aged at least 18 years old from both genders. Studies were included if it analysed at least one of the above-mentioned biomarkers. We excluded intervention studies with tocotrienol as we aimed at comparing effects of fatty acids only. Non-English language studies, reviews, proceedings, qualitative studies, mechanism studies as well as in-vivo and in-vitro studies were also excluded.

### 2.2 Data sources and search strategy

We systematically searched for relevant articles published in The Cochrane Central Register of Controlled Trials (CENTRAL), PubMed/MEDLINE and Scopus databases. We identified articles published up to 8^th^ June 2018. We also performed cross-reference of related studies and reviews to identify potential studies for inclusion. We searched using a strategy combining the Cochrane Highly Sensitive Search Strategy for identifying randomized trials in MEDLINE: sensitivity- and precision-maximizing version [23] with selected MeSH terms and free text terms relating to diabetes mellitus. We used the following combinations of keywords: (((((((("diabetes mellitus"(MeSH Terms) OR non-insulin dependent diabetes mellitus (Title/Abstract)) OR NIDDM(Title/Abstract)) OR glucose (Title/Abstract)) OR hyperglycaemia (Title/Abstract)) OR insulin (Title/Abstract)) OR HbA1c (Title/Abstract)) OR HOMA (Title/Abstract)) OR insulin resistance(Title/Abstract)) AND ((((((((((((palm oil(Title/Abstract) OR *Elaeis guineensis* (Title/Abstract)) OR palm olein (Title/Abstract)) OR palmitic acid (Title/Abstract)) OR palm kernel (Title/Abstract)) OR palm stearin (Title/Abstract)) OR oleic acid (Title/Abstract)) OR linoleic acid (Title/Abstract)) OR lauric acid (Title/Abstract)) OR myristic acid (Title/Abstract)) OR palmitoleic acid (Title/Abstract)).

### 2.3 Study selection

A pair of authors independently assessed the titles and abstracts of a defined set of articles. Each study was recorded as include, exclude or unclear. Full articles were retrieved for further assessment if they were recorded as include or unclear. Eligible studies were identified based on the inclusion criteria. Any discrepancies in the assessment were resolved by discussion leading to a consensus, with a third party serving as arbitrator if necessary.

### 2.4 Data extraction and risk of bias assessment

We designed a Data Extraction Form (DEF) for data collection from all potential studies. The DEF included information on study characteristics (type of study, presence of washout periods, study duration), participant characteristics (method of participant selection, inclusion criteria, exclusion criteria, demographics and co-morbidities), intervention characteristics (type of intervention, duration of intervention, diet composition, fatty acid composition of diet, percentage of energy from fats provided by the intervention diet, and the percentage of energy exchanged by the specific test fat), analysis and results (baseline characteristics, baseline clinical parameters such as body mass index (BMI), and fasting plasma glucose, and outcomes), compliance assessment (weight measurement, biomarkers of compliance measured) and source of funding. All authors independently extracted the data and any discrepancies were resolved by discussion. The characteristics of the included studies are outlined in Table 1 and S2 Table.

**Table 1:** Characteristics of included studies.

*Source (Country)*	Study Design	Duration of study (weeks)	Duration of wash out period, if applicable (weeks)	Intervention	Number of participants in intervention group	Number of participants in control group	Sex (% Men)	Age (years)	Baseline mean BMI (kg/m2)	Baseline fasting glucose (mmol/L)	Characteristic of participants (co-morbidities)
***Vega-Lopez et al*. *2006*, *USA*** [31]	Crossover study	7	None	Palm Oil/ Partially hydrogenated soybean oil/ Soybean oil/ Canola oil	15		33.33	63.9 (5.7)	26 (2.4)	4.6 (0.4)	Normal fasting glucose concentration.
***Sundram et al*. *2007*, *Malaysia*** [32]	Randomised crossover	4	None	Palm Olein/ Partially hydrogenated soybean oil	30		33.33	30 (8)	22 (4)	5.43 (0.29)	No adherence to any medications
***Karupaiah et al*. *2016*, *Malaysia*** [33]	Randomised double-blinded crossover	4	2	Palm Olein/ Soybean oil	17		47.06	23.4 (7.0)	25.1 (4.7)	4.70 (0.44)	No history of diabetes.
***Sun et al*. *2018*, *China*** [34]	Randomised double-blinded crossover	5	2	Palm Oil/ Olive Oil	100		53	40.29 (9.14)	22.19 (2.1)	<6.1	No personal or family history of diabetes.
***Filipou et al*. *2014*, *Malaysia*** [35]	Single-blind crossover	6	None	Palm Oil/ High Oleic Sunflower Oil	41		24.39	29.13 (7.6)	23 (3)	N/A	No history of diabetes, current use of insulin/glucose modulating medications.
***Mensink et al*. *2008*, *Netherlands*** [36]	Randomised double-blinded crossover	3	1	Palm Olein/ Rapeseed Oil	44		25	20 (1)	23.2 (2.94)	5.51 (0.44)	No history of glucosuria
***Rosqvist et al*. *2014*, *Sweden*** [37]	Randomised double-blinded controlled trial	7	N/A	Palm Oil/ Sunflower Oil	19	18	70.27	26.9 (4.08)	20.2 (4.7)	4.70 (0.44)	No history of diabetes.
***Thorlsrtrup et al*. *2011*, *Germany*** [38]	Randomised double-blinded crossover	3	None	Palm Olein/ Olive oil	32		100	29.6 (10.3)	22.9 (2.5)	5.33 (0.37)	No chronic diseases.

Abbreviations: BMI, body mass index; N/A, not available in the publication

### 2.5 Study risk of bias

We assessed the study quality of each included study using the COCHRANE guideline for assessment of systematic reviews [24] and the published guide on risk of bias assessment for crossover studies [27]. The studies were evaluated based on eight criteria: appropriate crossover design, randomised order of receiving treatment, carry-over effects, unbiased data, allocation concealment, blinding, incomplete outcome data and selective reporting. For each item, risk of bias was classified as ‘low risk’, ‘high risk, or ‘unclear risk’, with the last category indicating either lack of information or uncertainty over the potential for bias. Results were presented in a ‘Risk of bias’ summary (S1 Fig and S3 Table).

### 2.6 Grading of recommendations assessment, development, and evaluation (GRADE)

We evaluated the quality of evidence of the outcomes using the Grades of Recommendation, Assessment, Development and Evaluation Working Group (GRADE Working Group) framework [28]. The GRADE approach assesses the confidence in the effect estimates derived from the body of evidence (quality of evidence) by outcome and produce evidence profiles. The overall quality of evidence was presented in the form of ‘Summary of findings’ Table (S4 Table). The assessment of quality was based on five factors: risk of bias across all studies, indirectness, effect estimates, inconsistency amongst studies, imprecisions, and publication bias. Confidence in the estimate of each association was categorized into four levels, from very low to high.

### 2.7 Data synthesis and analysis

Data synthesis and analysis were carried out using Review Manager software (Rev Man) version 5.3 (Nordic Cochrane Centre, Cochrane Collaboration, Copenhagen). We standardised the values obtained from each of the included studies. Fasting plasma glucose and 2-hour post prandial glucose values were converted into millimole per litre. HOMA values were taken in percentages while fasting insulin values were converted into picomoles per litre. Other values such as interquartile ranges, confidence intervals were converted when necessary [29]. End of intervention values were obtained as mean and standard deviation from the respective groups. We did not take changes in the outcome values from baseline in crossover trials as there could be a carry-over effect.

Quantitative analysis were made between similar intervention and comparison oils, and qualitative assessment of the included studies were made when comparisons could not be performed. Mean differences were calculated for the different outcomes with same scales. We performed meta-analysis of end of intervention means and adopted a random effects model for the meta-analysis because of the heterogeneity of the effects across the included studies. Heterogeneity between studies was assessed with Cochran’s Q test (significant at P<0.10), and quantified with the *I*^2^ statistic (range from 0% to 100%) [30].

## 3. Results

### 3.1 Search results

We identified 1921 potentially eligible articles through our electronic databases search. A total of 1887 articles were excluded following titles and abstracts screening due to irrelevant studied population, intervention characteristics, comparator characteristics and outcomes. Thirty-four full text articles were then screened of which 26 articles were excluded for the following reasons: irrelevant interventions (twelve), irrelevant comparators (five), irrelevant outcomes (four), incomplete outcome data (three) and unclear source of fatty acids (two). Eight studies are included in this review [31–38]. Comparisons were made in between palm oil or palm olein with partially hydrogenated soybean oil (PHSO) [31,32], soybean oil [31,33] and olive oil [34,38], with two studies in each of the comparison. Comparisons of palm oil consumption with other vegetable oils could not be made for 2-hour post prandial glucose and HbA1C (no studies) in view of limited available evidence (one study) [35]. Our search PRISMA flowchart is presented in Fig 1.

We extracted descriptive data of all eight included trials [31–38] (Table 1, S2 Table). Only one study [37] was a parallel trial design while other studies were crossover study design, with half of them [33,34,36] applied one or two weeks of wash-out period. The intervention studies were conducted in Malaysia (three studies) [32,33,35], China (one study) [34], Netherland (one study) [36], Sweden (one study) [37], Germany (one study) [38] and USA (one study) [31]. Our review includes 333-participant data. The pooled mean age of all of the participants was 32.13 years old (standard deviation = 6.96 years) with total male participants of 47.15%. The studies mainly included young and middle-aged participants (20–40 years old) with the exception of one study [31]. All studies were conducted in healthy participants without any reported history of diabetes mellitus and with normal BMI. At baseline, the pooled mean BMI of all participants was 22.72 kg/m^2^ (standard deviation = 3.09).

Five vegetable oils/fat were included as comparators in the study, which comprised of PHSO, soybean oil, sunflower oil, canola oil, olive oil and rapeseed oil. For each study, the total percentage of energy contributed by the specified fatty acid was similar which was 15–20%. Only four studies [32–35] explained in detail the preparations of interventions. Each participant during the intervention period was given three-course meals during weekdays and provided test oil during weekends [32–35]. Compliance measurement using standard assessment for dietary fatty acids intervention (fatty acid profile) were done in only two studies [31,32], while most studies used food record or diary [33,34,36–38]. In addition, all studies monitored body weight and maintain usual physical activities. Rosqvist and colleagues [37] excluded two individuals due to unexplained weight loss during intervention, while the rest of the studies were able to keep body weight within the desired range.

### 3.2 Fasting plasma glucose

**Fig 2:**
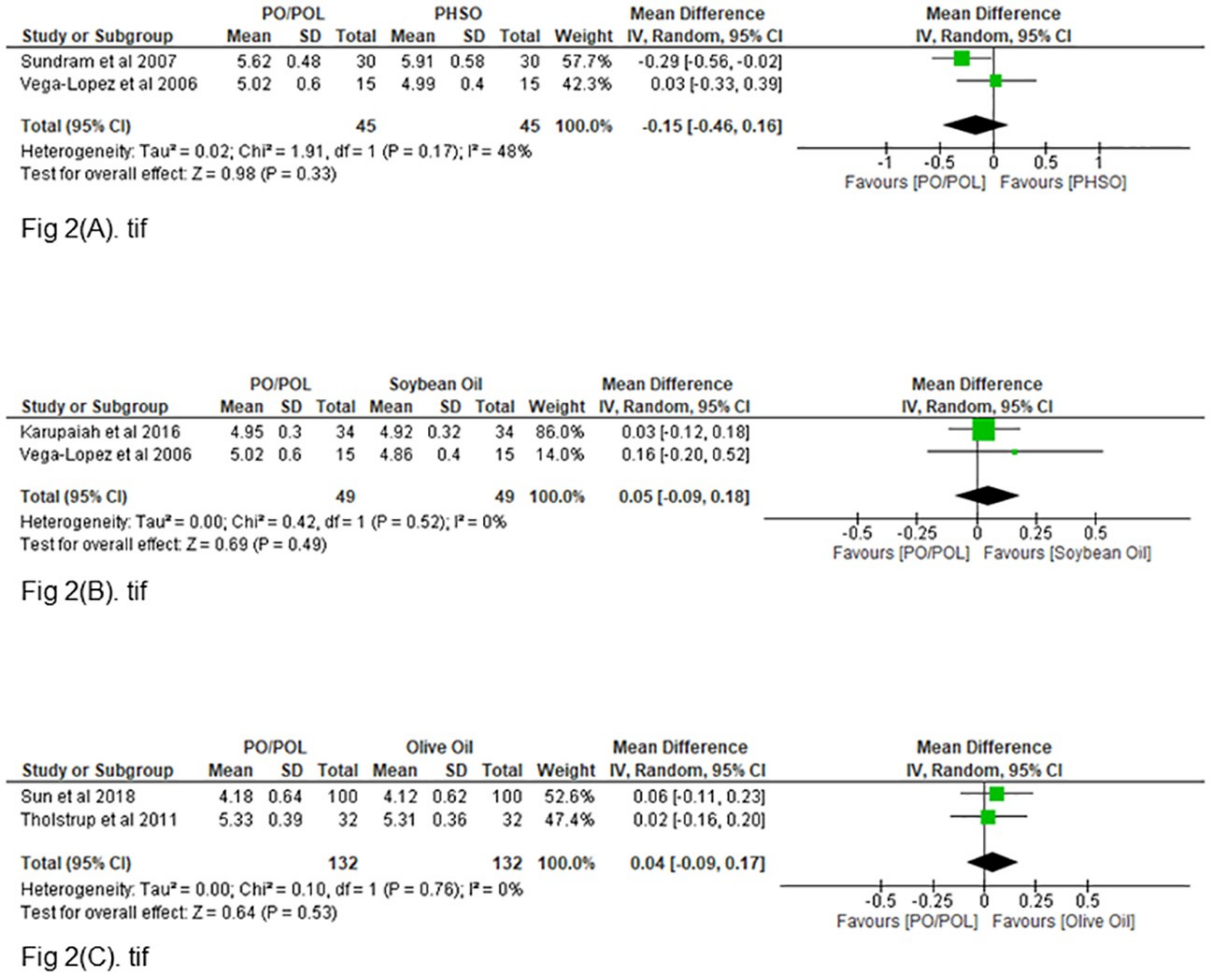
**(A)**: Pooled mean difference of fasting plasma glucose for palm oil- and PHSO diets (mmol/l) **(B)**: Pooled mean difference of fasting plasma glucose for palm oil- and soybean oil diets (mmol/l) **(C)**: Pooled mean difference of fasting plasma glucose for palm oil- and olive oil diets (mmol/l).

All eight included studies evaluated the changes in fasting plasma glucose for palm oil consumption as compared to other vegetable oils. The analysis for palm oil or palm olein rich diets compared to PHSO diets [31,32], soybean oil diets [31,33] and olive oil diets [34,38] are presented in the Fig 2(A), 2(B) and 2(C) respectively. Two studies were included for each of the comparisons. Non-significant effects in fasting plasma glucose were seen when compared to PHSO diet [-0.15mmol/L (95%CI = -0.46,0.16) P = 0.33; I^2^ = 48%], soybean oil diet [0.05mmol/L (95%CI = -0.09,0.18) P = 0.49, I^2^ = 0%] and olive oil diet [0.04mmol/L (95%CI = -0.09,0.17) P = 0.53, I^2^ = 0%]. Non- significant effects were also seen when compared with canola oil diet [MD = 0.12mmol/L (95%CI = -0.28,0.52)], rapeseed oil diet [MD = -0.03mmol/L (95%CI = -0.21, 0.15)], high oleic sunflower oil diet [MD = 0.00mmol/L (95%CI = -0.14,0.14)] and sunflower oil diet [MD = -0.20mmol/L (95%CI = -0.43,0.03)]. Subgroup analyses or publication bias tests were not performed (<10 studies).

### 3.3 Fasting insulin

**Fig 3:**
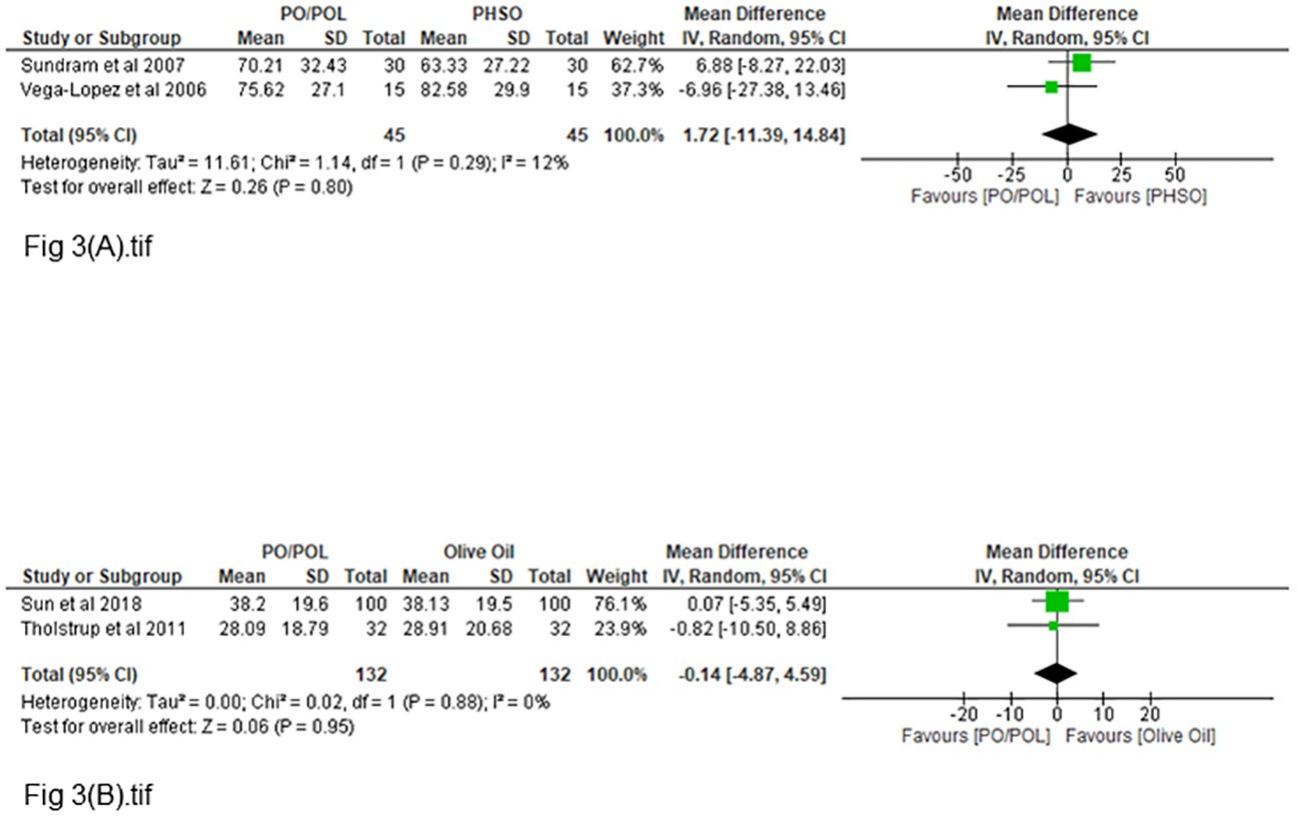
**(A)**: Pooled mean difference of fasting insulin for palm oil- and PHSO diets (pmol/l) **(B)**: Pooled mean difference of fasting insulin for palm oil- and olive oil diets (pmol/l).

A total of six studies [31,32,34–36,38] reported fasting insulin outcomes for comparison of palm oil and other vegetable oil diet. Two studies were included for each of the comparison between palm oil or palm olein rich diets with PHSO diets [31,32] and olive oil diets [34,38]. The analysis for palm oil or palm olein rich diets compared to PHSO diets and olive oil diets are presented in the Fig 3(A) and 3(B) respectively. There were insignificant effects on fasting insulin when compared to PHSO diet [1.72pmol/L (95%CI = -11.39,14.84) P = 0.80, I^2^ = 12%] and olive oil diet [-0.14pmol/L (95%CI = -4.87,4.59) P = 0.95, I^2^ = 0%]. Non-significant effects were also seen when compared with soybean oil diet [MD = 6.38pmol/L (95%CI = -12.35,25.11)], canola oil diet [MD = 8.82pmol/L (95%CI = -9.83,27.47)], rapeseed oil diet [MD = -1.60pmol/L (95%CI = -10.12,6.92)], high oleic sunflower oil diet [MD = -1.39pmol/L (95%CI = -15.17,12.39)] and sunflower oil diet [MD = -5.42pmol/L (95%CI = -40.20,29.36)]. Subgroup analyses or publication bias tests were not performed (<10 studies).

### 3.4 Homeostatic model assessment-insulin resistance (HOMA)

Four studies [31,34,35,37] assessed the changes in HOMA for consumption of palm oil- or palm olein-rich diet as compared to other vegetable oil diets. Palm oil- or palm olein-rich diets were compared to diets with PHSO, soybean oil, canola oil, sunflower oil, olive oil and high oleic acid sunflower oil [31,34,35,37]. There were insignificant effects in HOMA when compared to PHSO diet [MD = -0.20% (95%CI = -0.88,0.48)], soybean oil diet [MD = 0.31% (95%CI = -0.30,0.92)], canola oil diet [MD = 0.36% (95%CI = -0.23,0.95)], sunflower oil diet [MD = -0.23% (95%CI = -0.49,0.03)], olive oil diet [MD = 0.11% (95%CI = -0.06,0.28)], and high oleic acid sunflower oil diet [MD = -0.01 (95%CI = -0.24,0.22)].

### 3.5 2-hour post prandial glucose

Only one study measured 2-hour post prandial glucose [35] and reported no significant difference [MD = 0.00mmol/L (95%CI = -0.19,0.19)] after consumption of palm oil compared to high oleic sunflower oil.

### 3.6 Risk of bias assessment

We judged the risk of possible bias present in the included studies according to the eight criteria incorporated in Cochrane’s tool for assessing risk of bias. We presented the summary of risk of bias assessment of the included studies in S1 Fig and S3 Table. Random sequence generation was assessed to be of low risk of bias in five studies [31,33–35,37], and of unclear risk of bias [32,36,38] in the remaining three as they did not clearly state the method of random sequence generation. When assessing the allocation concealment of the included studies, only one study [37] had low risk of bias while the other six studies [31–36,38] did not clearly state the method of allocation of treatment groups. Performance bias was assessed to be low risk of bias in all studies. All studies reported low risk of bias for attrition bias domain except for one study [35] due to high dropout rate. Reporting bias domain had only one study [35] with low risk of bias. One study was considered high risk due to reporting bias as the study did not reported few of their pre-specified outcomes (C-peptide, hsCRP, IL-6, TNF α) [34], while the remaining six studies [31–33,36–38] were unclear. When assessing the appropriateness of the crossover design, we evaluated four studies [31,32,35,38] to be high risk of bias because of unavailability of wash-out periods between interventions. Therefore, we evaluated the same four studies [31,32,35,38] without a washout period to potentially have carry-over effects. All crossover studies had low risk of bias for unbiased data as all analysed the outcomes at different time points.

### 3.7 GRADE assessment

We graded the evidences for changes in fasting glucose for the consumption of palm oil or palm olein with PHSO, olive oil and soybean oil as low grade. The evidences were downgraded in view of limitations in risk of bias (high risk of bias in the studies’ method), indirectness (study populations were among healthy and young to middle age participants) and imprecision (each of the evidence had wide and non-significant confidence intervals). Low quality of evidence suggests that the effect estimate is limited and that the true effect may be substantially difference from the effect estimate.

The quality of evidence for changes in fasting insulin for palm oil-rich diet with PHSO- and olive oil- rich diet were graded as very low and low quality of evidence respectively. We downgraded three levels in the comparison with PHSO-rich diet because of limitations in risk of bias (high risk of bias in method), indirectness (study populations were among healthy and young to middle age participants) and imprecision (very wide confidence interval). The evidence in the comparison with olive oil-rich diet was also downgraded two levels due to limitations in risk of bias (high risk of bias in method) and imprecision (wide confidence interval).

## 4. Discussion

### Principal findings

Our findings reveal that there is very low to low grade of evidence for the changes in fasting glucose and insulin for the consumption of palm oil or palm olein as compared to other vegetable oils (olive oil, PHSO, soybean oil) in healthy, young to middle-aged individuals. There was insufficient evidence to synthesize its association with HOMA, 2-hour post prandial glucose and also HbA1c.

Previous animal studies indicate that TFA might impair fat cell membrane-fluidity and insulin sensitivity [39,40]. Meta-analysis of seven randomized controlled trials showed that an increase in TFA intake from 2.5% to 7.8% of total energy intake did not lead to any significant changes in circulating glucose and insulin concentration [41]. Similarly, clinical studies failed to show any significant changes in insulin sensitivity with 5–9% of total energy intake TFA consumption compared with SFA consumption [42]. In our study, despite the quantity of TFA in PHSO were 3.2% [32] and 4.15% [31], higher than average amount consumed in United States [43], there was no significant association revealed for fasting glucose, HOMA and insulin.

Existing evidence suggests that lowering risk of type 2 diabetes mellitus is associated with consumption of omega-6 PUFA and total PUFA, in addition to omega-3 PUFA [44]. PUFA intake increases membrane fluidity which augments insulin sensitivity and helps to counter toxicity of tissue free fatty acids [18]. Imamura and colleagues [45] reported significant lower levels of fasting glucose (-0.04 mmol/L; 95% CI -0.07,-0.01; p = 0.028) and HOMA (-4.1%; 95% CI -6.4, -1.6; p<0.05) after replacement of SFA with PUFA. However, in view of the small number of studies and participants, non-significant effects were seen for fasting glucose in our study.

One of the major components of olive oil is oleic acid which belongs to the class of MUFA [46]. However, one of the unique qualities of palm oil is that its oleic acid is predominantly at the sn-2 position in major triacylglycerols that makes palm oil acts more like MUFA than typical SFA [47]. Previous review showed that MUFA consumption did not significantly influence fasting glucose but did improve both HbA1c and HOMA in comparison to SFA [45]. We reported similar finding only in fasting glucose, but not in HOMA or HBA1c when comparing olive oil and palm oil in two studies [34,38], in view of small number of studies and participants.

### Study strengths and limitations

A major limitation of our study is the limited number of relevant studies with small sample sizes which reduced the strength of the evidence. Furthermore, the population in the included studies were healthy and mainly of young to middle-aged adults, thus most individual studies did not report statistically significant effects on our interest endpoints. Imamura and colleagues had shown significant lowered glucose, HbA1c, C-peptide and HOMA when replacing SFA with PUFA on diabetic population [45]. Six of the included studies [31,33,34–38] did not have glucose metabolism biomarkers as their primary outcomes. Therefore, lack of data to estimate the effect on insulin sensitivity by gold standard techniques or on insulin or glucose metabolism after a meal challenge, which may be more informative for metabolic status and predicting diabetes risk. The duration of the included studies was short (three to seven weeks), thereby limiting the long-term effects evaluation of these biomarker. However, it is still possible to influence insulin sensitivity within 4 weeks in healthy individuals by changing fat quality alone [48,49]. Four of the crossover studies [31,32,35,38] did not apply any washout periods which may potentially have a carryover effect to the outcome of interest. A frequent recommendation for washout period is to be at least 5 times the half-life of the treatment with the maximum half-life in the study [50], therefore, 2 weeks duration of washout period is sufficient to minimize any carryover effects [51]. In view of both dietary habit and genetics are an important determinant of diabetes mellitus, another limitation is the heterogeneity of study population and the diets. Current review included one study [31] that was conducted in the United States where consumption of soybean oil is higher [52], while other studies were conducted in Asia (three in Malaysia and one in China) and European countries (Netherland, Sweeden, Germany) where palm oil is the main edible vegetable oil consumed [2]. Furthermore, structural equation modelling revealed that genetic influences accounted for a significant portion of the total variance in total energy, macronutrients, minerals, and vitamins [53]. Although, all the studies had isocaloric diet and only differed in bond configuration in fatty acids, however, all the fat were incorporated into variety of meals. This can effect on the gastric emptying, and therefore, glucose and insulin responses [54].

This study has several strengths. To our knowledge this is the first review to evaluate the effect of palm oil intake in relation to glucose metabolism biomarkers whereby previous studies mostly focused on cardiovascular disease risks, especially lipids. Our review only assessed vegetable oils, while previous studies evaluate different sources of food and analysed according to predominant fatty acids. However, the type of fatty acid and its stereospecificity in triacylglycerol molecular species largely determines the physical behavioural of dietary fats, specifically in early metabolic processing and postprandial clearance, therefore, has different effects in glucose biomarkers [55]. We assessed the outcome estimates with GRADE to facilitate assessment of strength of evidence. We systematically identified the potentially relevant articles through the electronic databases, complemented by manual searches of reference lists of relevant articles.

### Recommendations

We recommend that more longitudinal population-based studies are performed and reporting of the trial according to the CONSORT statement guidelines. The test diets in current controlled feeding trials are typically isocaloric and differ only in dietary fat quality. However, such studies are usually small and short in duration, and thus can only evaluate intermediate endpoints. Therefore, high quality trials with big sample sizes and longer study periods are warranted. It is also important that studies compare effects of vegetable oils on type 2 diabetes patients, and not only on healthy participants.

## 5. Conclusion

In conclusion, current evidence on the effects of palm oil consumption on biomarkers of glucose metabolism is poor and limited to only healthy participants. We conclude that little or no additional benefit will be obtained by replacing palm oil with other oils rich in mono or polyunsaturated fatty acids for changes in glucose metabolism.

## Supporting information

S1 Fig Risk of bias summary. (XLSX)

S1 Table PRISMA 2009 checklist. (DOC)

S2 Table Characteristics of included studies. (DOCX)

S3 Table Detailed risk of bias assessment. (DOCX)

S4 Table GRADE table. (DOCX)
