# Supplementary material for: Effects of palm oil consumption on biomarkers of glucose metabolism: A systematic review
Source: PLoS One. 2019 Aug 15;14(8):e0220877. doi: 10.1371/journal.pone.0220877 (PMC6695104; doi:10.1371/journal.pone.0220877)
Supplement: S2 Table — (DOCX) [file pone.0220877.s003.docx]

**S2 Table. Characteristics of included studies**

| Trials | Vega-Lopez et al. (2006) [31] | Sundram et al. (2007) [32] | Karupaiah et al. (2016) [33] | Sun et al. (2018) [34] | Filipou et al. (2014) [35] | Mensink (2008) [36] | Rosqvist et al. (2014) [37] | Thorlstrup et al. (2011) [38] |
| --- | --- | --- | --- | --- | --- | --- | --- | --- |
| **Outcomes measured** | FPG, fasting insulin, HOMA | FPG, fasting insulin | FPG | FPG, fasting insulin, HOMA | FPG, fasting insulin, HOMA, 2HPP | FPG, fasting insulin | FPG, HOMA | FPG, fasting insulin |
| **Weight loss** | No | No | No | No | No | No | No | No |
| **Delivery Vehicle** | Meal | Meal | Mayonnaise | Meal | Meal | Margarines, cookies, muffins, chocolate paste and chips. | Muffins | Buns and cakes |
| **Compliance assessment** | Plasma FA | Plasma TG FA | DR | DR | Lipids | DR | DR | DR |
| **Percentage of energy contributed by test fat (%)** | 20 | 20 | 20 | 18 | 20 | 15 | N/A | 17 |
| **Micronutrient intake SFA/MUFA/PUFA % of energy in intervention** | 15/11/4 | 14/14/4 | 11/12/7 | N/A | 11/11/4 | 15/16/6 | 12/10/4 | 14/15/6 |
| **Micronutrient intake SFA/MUFA/PUFA % of energy in control** | Canola- 6/15/9 Soybean- 7/8/13  PHSO- 9/10/8 | 9/12/6 | 8/9/12 | N/A | 4/17/4 | 12/19/6 | 13/11/5 | 9/20/6 |
| **Macronutrient intake Fat/CHO/Prot % of energy in intervention** | 30/52/18 | 30/54/15 | 30/54/15 | 31/58/11 | 27/55/18 | 40/45/15 | 32/50/14 | 36/49/13 |
| **Macronutrient intake Fat/CHO/Prot % of energy in control** | Canola- 32/53/15  Soybean- 28/56/16  PHSO- 30/53/15 | 30/54/15 | 30/54/15 | 28/61/11 | 27/55/18 | 40/44/15 | 35/45/14 | 35/51/12 |
| **Source of Funding** | US NIH, USDA | MPOB | Kewpie Corporation, Japan | MPOB, Research Funds for the Central Universities | MPOB, PhD studentship awarded to A Filippou from King’s College London | Cargill Refined Oils Europe | Swedish Research Council | N/A |

Abbreviations: FPG fasting insulin; HOMA homeostatic model assessment; 2HPP 2 hour post-prandial; FA fatty acids, PL Phospholipid, DR Diet records, TG triaclyglyceride, CHO carbohydrate; SFA saturated fatty acids; MUFA monounsaturated fatty acid; PUFA polyunsaturated fatty acids; N/A not available; MPOB Malaysian Palm Oil Board; US NIH United states National Institute of Health.
