# Supplementary material for: Effects of palm oil consumption on biomarkers of glucose metabolism: A systematic review
Source: PLoS One. 2019 Aug 15;14(8):e0220877. doi: 10.1371/journal.pone.0220877 (PMC6695104; doi:10.1371/journal.pone.0220877)
Supplement: S3 Table — (DOCX) [file pone.0220877.s004.docx]

**S3 Table: Detailed risk of bias assessment.**

|  | Vega-Lopez et al 2006 [31] | Sundram et al 2007 [32] | Karupaiah et al. 2016 [33] | Sun et al 2018 [34] | Filipou et al. 2014 [35] | Mensink 2008 [36] | Rosqvist et al 2014 [37] | Thorlstrup et al 2011 [38] |
| --- | --- | --- | --- | --- | --- | --- | --- | --- |
| **Randomized treatment order (selection bias)** | Randomized according to Latin-square design (LOW) | Method use for randomization was not described (UNCLEAR) | Randomized according to parity for gender, age and plasma TC levels. (LOW) | Allocated using random table method (LOW) | Allocated by a computer program. (LOW) | Allocated by gender then randomly assigned. (UNCLEAR) | Allocated by drawing lots with a fixed block size of 4 (LOW) | Method use for randomization was not described (UNCLEAR) |
| **Allocation concealment (selection bias)** | Not stated (UNCLEAR) | Not stated (UNCLEAR) | Not stated (UNCLEAR) | Not stated (UNCLEAR) | Not stated. (UNCLEAR) | Not stated (UNCLEAR) | Allocated was only known by one researcher and concealed from other investigators (LOW) | Not stated (UNCLEAR) |
| **Blinding of participants and personnel (performance bias)** | Participants and researchers were blinded (LOW) | Participants were blinded using labelling (LOW) | Researchers and participants were blinded to the intervention using labelling (LOW) | Double-blinding, which individuals blinded not stated (LOW) | Participants were blinded to the treatment. (LOW) | Participants and researchers were blinded using letter-coded (LOW) | Participants and researchers were blinded using labelling (LOW) | Double-blinding, which individuals blinded not stated (LOW) |
| **Incomplete outcome data (attrition bias)** | No dropout (LOW) | 2 participants withdrew (LOW) | 2 participants withdrew (LOW) | 20 participants dropout, 15% of incomplete data (LOW) | 12 participants withdrew. 22% of incomplete data (HIGH) | 5 participants were excluded (LOW) | 2 participants withdrew (LOW) | 2 participants withdrew (LOW) |
| **Selective outcome reporting (reporting bias)** | CIinical trial registry was not available (UNCLEAR) | CIinical trial registry was not available (UNCLEAR) | CIinical trial registry was not available (UNCLEAR) | CIinical trial registry is available, not all pre-specified outcomes were reported (HIGH) | CIinical trial registry is available, all pre-specified outcomes were reported (LOW) | CIinical trial registry was not available (UNCLEAR) | CIinical trial registry was not available (UNCLEAR) | CIinical trial registry was not available (UNCLEAR) |
| **Other bias**  **Carry-over effect,** | No washout period (HIGH) | No washout period (HIGH) | 2 weeks washout period (LOW) | 2 weeks washout period (LOW) | No washout period (HIGH) | 1 week washout period (LOW) | Parallel study (LOW) | No washout period (HIGH) |
