# Supplementary material for: Effects of palm oil consumption on biomarkers of glucose metabolism: A systematic review
Source: PLoS One. 2019 Aug 15;14(8):e0220877. doi: 10.1371/journal.pone.0220877 (PMC6695104; doi:10.1371/journal.pone.0220877)
Supplement: S4 Table — (DOCX) [file pone.0220877.s005.docx]

**S4 Table. GRADE**

| **Does consumption of palm oil-rich diet negatively affect glucose biomarkers as compared to other edible vegetable oils?** | | | | | | | | | |
| --- | --- | --- | --- | --- | --- | --- | --- | --- | --- |
| **Certainty assessment** | | | | | | | **Summary of findings** | | |
| **№ of participants (studies)** | **Risk of bias** | **Inconsistency** | **Indirectness** | **Imprecision** | **Publication bias** | **Overall certainty of evidence** | **No of participants *** | | **Anticipated absolute effects#** |
|  |  |  |  |  |  |  | **PO** | **Other Vegetable Oil** | **Effect estimate (mean difference [95% CI])** |
| **Changes in fasting glucose between palm oil and PHSO** | | | | | | | | | |
| 90 (2 RCTs) | serious | not serious | serious | serious | Not assessed | ⨁◯◯◯ LOW^a^ | 45 | 45 | -**0.15** (-0.46, 0.16 )mmol/L |
| **Changes in fasting glucose between palm oil and soybean** | | | | | | | | | |
| 98 (2 RCTs) | serious | not serious | serious | serious | Not assessed | ⨁◯◯◯ LOW^a^ | 49 | 49 | **0.05** (-0.09, 0.18)mmol/L |
| **Changes in fasting glucose between palm oil and olive oil** | | | | | | | | | |
| 264 (2 RCTs) | serious | not serious | serious | serious | Not assessed | ⨁◯◯◯ LOW^a^ | 132 | 132 | **0.04** (-0.09, 0.17)mmol/L |
| **Changes in fasting insulin between palm oil and PHSO** | | | | | | | | | |
| 90 (2 RCTs) | serious | not serious | serious | very serious | Not assessed | ⨁◯◯◯ VERY LOW^b^ | 45 | 45 | **1.72**  (-11.39, 14.84)pmol/L |
| **Changes in fasting insulin between palm oil and olive oil** | | | | | | | | | |
| 264 (2 RCTs) | serious | not serious | serious | serious | Not assessed | ⨁◯◯◯ LOW^c^ | 132 | 132 | **-0.14**  (-4.87 , 4.59)pmol/L |

**PO = palm oil, POL = palm olein, PHSO = partially hydrogenated soybean oil**

***Number of participants** presented as the total number of participants in the control group (comparison) and in the intervention group.

**^#^Anticipated absolute effects** presented as mean difference (95% confidence interval)

^a^ Downgraded three levels due to limitations in risk of bias (high risk of bias in method), indirectness (population were among healthy, young to middle aged participants) and imprecision (wide and non-significant confidence interval)

^b^ Downgraded three levels due to limitations in risk of bias (high risk of bias in method), indirectness (population were among healthy, young to middle aged participants) and imprecision (very wide confidence interval)

^c^ Downgraded three levels due to limitations in risk of bias (high risk of bias in method), indirectness (population were among healthy, young to middle aged participants) and imprecision (wide confidence interval)
